# Identifying the topology of signaling networks from partial RNAi data

**DOI:** 10.1186/s12918-016-0301-4

**Published:** 2016-08-01

**Authors:** Yuanfang Ren, Qiyao Wang, Md Mahmudul Hasan, Ahmet Ay, Tamer Kahveci

**Affiliations:** 1 Department of Computer & Information Science & Engineering, University of Florida, Gainesville, 32611 FL USA; 2 Department of Biology & Mathematics, Colgate University, Hamilton, 13346 NY USA

**Keywords:** Signal transduction networks, Network inference, RNAi data, Missing data

## Abstract

**Background:**

Methods for inferring signaling networks using single gene knockdown RNAi experiments and reference networks have been proposed in recent years. These methods assume that RNAi information is available for all the genes in the signal transduction pathway, i.e., complete. This assumption does not always hold up since RNAi experiments are often incomplete and information for some genes is missing.

**Results:**

In this article, we develop two methods to construct signaling networks from incomplete RNAi data with the help of a reference network. These methods infer the RNAi constraints for the missing genes such that the inferred network is closest to the reference network. We perform extensive experiments with both real and synthetic networks and demonstrate that these methods produce accurate results efficiently.

**Conclusions:**

Application of our methods to Wnt signal transduction pathway has shown that our methods can be used to construct highly accurate signaling networks from experimental data in less than 100 ms. The two methods that produce accurate results efficiently show great promise of constructing real signaling networks.

## Background

Cells respond to external stimuli often initiated by external signaling molecules such as steroid hormones or growth factors. This response is tightly controlled by complex protein-protein interaction networks, namely, signal transduction pathways [1]. When an external molecule binds to a specific *receptor* molecule located in the cell membrane or inside the cell, the receptor undergoes a conformational change and triggers a chain of signaling events to propagate the external signal inside the cell. As the appropriate response to the external stimuli, the chain of biochemical reactions culminate in the activation or suppression of a target protein (or a set of proteins) known as the *reporter* protein.

Signaling networks are vital for proper functioning of cells as they govern key cellular processes. For instance, Mitogen-activated protein kinase (MAPK) signaling network is involved in the regulation of cellular proliferation, differentiation, mitosis, survival, and apoptosis [2, 3]. Any disruption in signal transduction in cells leads to a number of disorders such as cancer, Alzheimer’s, Parkinson’s, and kidney and cardiovascular disease [4–7]. It is paramount that we study the topology of the signaling networks to gain insights into how cells respond to external stimuli, how its deviation results in various diseases and how the cells respond to treatments.

Experimental methods such as yeast-two hybrid, RNA interference (RNAi) give us information about the signaling events inside the cells. In the RNAi experiment [8], mRNA levels of a predetermined set of genes are artificially knocked down [8, 9]. For each gene, the effect of the knockdown is measured in the reporter genes. The role of the knocked down gene in the signal transduction pathway is inferred by comparing the responses of RNAi treated and wild type cells [10, 11]. If the response deviates greatly in the RNAi treated cells compared to the wild type, it shows that the knocked down gene plays an important role in signal transduction from the receptor to the reporter.

Single gene knockdown RNAi experiment gives insight about the importance of a single gene in signal transduction from receptor to reporter gene. However, constructing the complete network topology from RNAi experiments is computationally challenging [12]. To alleviate the computational cost, many computational methods have been developed that use available experimental data such as gene expression, RNAi knock down assay and protein-protein interaction networks [13–15]. These methods often employ Bayesian networks, probabilistic Boolean networks, combinatorial optimization methods and differential equation models [15–21]. Some inference algorithms start with a network topology called the *reference* network. These methods assume that the network to be constructed is similar (few network edit operations away) to the reference network [20–23]. The methods that utilize prior knowledge construct accurate network topology faster than methods that do not.

Signaling Network Constructor (SiNeC) [20] is an algorithm that infers signaling networks using a reference network and RNAi data. SiNeC starts from the signaling network of a reference organism, makes minimum number of interaction addition or deletion to this reference network so that it satisfies the RNAi data (or RNAi constraints) of the target organism. SiNeC assumes that the RNAi experimental data is available for all the genes in the network. However, RNAi experiments are often noisy, and there are usually genes that the RNAi data is not collected [24]. *Therefore, the development of network construction methods for incomplete RNAi experimental data is at most importance.*

Network construction using a reference network and complete RNAi data is NP-Complete [20]. If RNAi data is missing for a subset of genes, that further increases the complexity of the problem. Assume that there are *n* genes for which RNAi data is missing. Note that each of these genes can be either critical for signal transduction from receptor to reporter genes, or noncritical, i.e., each gene has two possibilities. Therefore, for *n* missing genes, an optimal solution must evaluate all 2^*n*^ possible configurations to compute the correct values for the missing genes. It is impractical to evaluate all 2^*n*^ constraint configurations since exhaustive method will fail as *n* increases.

### Our contributions

In this article, we construct signaling networks using incomplete/ missing RNAi data. We design and develop two iterative network construction algorithms namely the *holistic optimization* and the *prioritized optimization* algorithms to infer signaling networks. Assume that there are *n* genes with missing RNAi data. Holistic optimization evaluates each of these genes one by one to decide if it is critical or noncritical, leading to *O*(*n*^2^) constraint combinations. Prioritized optimization lowers the number of constraint combinations by exclusively setting each gene as critical and combining the genes that yield networks with the same distance to the reference network (more on *distance* in Section ‘Preliminary terms’) in subsets of genes. This divides the set of *n* unknown genes to *k* subsets of mutually exclusive genes where each subset is of size *n*_*i*_ ($\sum _{i=1}^{k} n_{i} = n$). In each iteration, prioritized optimization evaluates only the genes in a subset to see if it’s critical or noncritical, thus leading to only $O(\sum _{i=1}^{k} n_{i}^{2})$ iterations. We also develop a node ordering algorithm named *TopSoG* that takes causality into account and both holistic and prioritized optimization algorithms employ it as a subroutine.

We evaluate our methods using both synthetic and real signaling network dataset. To compare the performance with the gold standard, we also implement an exhaustive algorithm that evaluates all subsets of the genes with missing RNAi data and infers the network with the closest distance to the reference network. We found that the proposed methods run much faster than the exhaustive algorithm and produce the same accuracy levels in their inferred networks. For instance, it takes less than 100ms for our method to reconstruct highly accurate Wnt signaling networks for different organisms. We also evaluate our methods using synthetic networks by varying a broad spectrum of parameters, such as the number of genes with missing RNAi data, the number of nodes in the network and the amount of deviation between the reference and the target network to be constructed. We found our methods to be robust as they produced highly accurate networks in all these scenarios.

The organization of the rest of the paper is as follows. In Section ‘Method’, we formally define the problem and propose two algorithms to solve it. We present the results of our extensive experiments in Section ‘Results and discussion’ and conclude the paper in Section ‘Conclusions’.

## Method

In this section we present two novel methods we developed to solve the signaling network construction problem. First, we present the key terms used in our method. Then, we briefly explain the SiNeC algorithm. Next, we describe our two methods in detail, holistic optimization algorithm and prioritized optimization algorithm. Last, we explain our new sorting algorithm ToPSoG for the critical genes.

### Preliminary terms

We start by introducing the key terms that will help present our method. First, we introduce an important concept, critical and noncritical genes in a network with a receptor and reporter gene pair.

#### **Definition 1**

Assume that we are given a directed network *G*=(*V*,*E*) with receptor gene *v*_*s*_ and reporter gene *v*_*t*_, we say that a gene *v*∈*V* is a critical gene if there is no path from *v*_*s*_ to *v*_*t*_ that does not contain *v*. Otherwise, it is a noncritical gene.

**Fig. 1:**
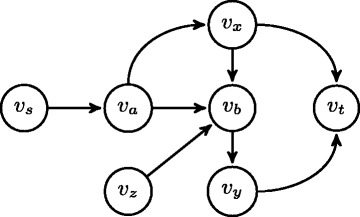
An hypothetical signaling network. Nodes *v*_*s*_ and *v*_*t*_ are the receptor and reporter genes. Nodes *v*_*a*_ and *v*_*b*_ are constrained to be critical genes

A simple example in Fig. 1 clarifies this. In this figure, node *v*_*a*_ appears on all the paths from *v*_*s*_ to *v*_*t*_. Thus, only node *v*_*a*_ is the critical node. Single gene knockdown RNAi experiments discover if a gene is important during the transmission of a signal from a receptor to a reporter gene. Let us denote the RNAi experiment result on the *i*th gene with an indicator variable *c*_*i*_. If a signal is unable to reach to the reporter gene from the receptor gene after the *i*th gene is knocked down, the variable *c*_*i*_=1. Otherwise, *c*_*i*_=0. If the RNAi experiment for the *i*th gene is missing, we set *c*_*i*_=−1. We call such genes as *unknown genes* in the rest of the paper. Suppose we want to construct a network with *l* genes, we represent the RNAi constraints imposed on all these genes with a vector of variables *C*=(*c*_1_,*c*_2_,…,*c*_*l*_). Following definition clarifies how to impose the RNAi constraints on a given network’s topology.

#### **Definition 2**

CONSISTENT NETWORK Consider a directed network *G*=(*V*,*E*) with a receptor and reporter gene pair, and the RNAi constraints *C* imposed on the set of genes. We say that *G* is consistent with *C* if ∀*v*_*i*_ is a critical gene when *c*_*i*_=1, or *v*_*i*_ is a noncritical gene when *c*_*i*_=0.

Notice that in Definition 2 above, only critical and noncritical genes are imposed rules. For unknown genes *c*_*i*_=−1, they can be either critical or noncritical. Next, we introduce another notation which is needed to define our problem.

#### **Definition 3**

DISTANCE BETWEEN TWO NETWORKS Assume that we are provided with two networks built on the same set of genes, *G*_1_=(*V*,*E*_1_) and *G*_2_=(*V*,*E*_2_). We denote the set difference and set cardinality with operators “ ∖” and “ ∣.∣” respectively. We define the distance between *G*_1_ and *G*_2_ as:

$$dist(G_{1},G_{2}) = \mid E_{1} \backslash E_{2} \mid + \mid E_{2} \backslash E_{1} \mid $$

In what follows we formally define the signaling network construction problem.

#### **Definition 4**

SIGNALING NETWORK CONSTRUCTION Assume that we are given a reference network *G*_*R*_=(*V*,*E*_*R*_) with respect to *v*_*s*_ and *v*_*t*_, and also a vector of RNAi constraints *C*. The problem is to construct a network *G*=(*V*,*E*) which is consistent with *C* and the distance *dist*(*G*,*G*_*R*_) to the reference is minimum.

It is important to note that Definition 4 conjectures that the topology of the reference network is close to that of target network. When reference networks from phylogenetically close organisms are available, this conjecture has already been shown to obtain accurate results [20].

Next, we present two novel algorithms we have developed for the problem as defined above. Both algorithms apply a hill climbing strategy. They first start from an initial configuration of constraints. Then they gradually update their constraints. Since we observe that there are usually a few critical genes in real signaling networks, in the initial configuration, for all *c*_*i*_=−1 (i.e., missing data), we set *c*_*i*_=0 (noncritical).

### Overview of the SiNeC algorithm

Before introducing our method, we first take a small detour to briefly summarize the SiNeC algorithm, which is necessary to better understand our method. SiNeC is a recent network inference algorithm which uses a given reference network and the RNAi data to construct the target network [20]. It however assumes that the RNAi constraints for all genes are known. In this paper, we develop algorithms that utilize SiNeC and deal with the missing RNAi data problem.

Briefly, SiNeC works in three steps: (i) It first estimates the order of critical genes in which the signal is propagated from the receptor to the reporter genes. SiNeC uses the Sloan algorithm [25] to generate a putative ordering. The Sloan algorithm assigns a priority value to each node based on its degree and its distance to the end node. It removes the node with highest priority and updates the priority of remaining nodes. It continues this process until all the nodes are processed. This greedy strategy results in an ordering which imposes that every path from receptor to reporter should pass critical genes in that order. (ii) SiNeC then deletes edges that conflict with the ordering of critical genes. If there is a path of noncritical genes between two nonconsecutive critical genes, a signal is still reachable without traversing through the intermediate critical genes. SiNeC deletes all these edges with minimum number of edge deletions to make the network consistent with the ordering of critical genes found by Sloan algorithm. (iii) SiNeC inserts some missing edges to make the reference network satisfy the experimental RNAi constraints. It inserts an edge if one of the following cases happens: 1) No path exits between two consecutive critical genes, or 2) At least a noncritical gene exits on all the paths between two consecutive critical genes, i.e, eventually making it a critical gene. For any further and detailed information, the interested readers can refer to Hashemikhabir et al. [20].

### Holistic optimization algorithm

Holistic optimization algorithm starts to construct the network topology with each unknown gene setting to noncritical. Then it iteratively tries to alter the constraint of one unknown gene at a time from noncritical to critical. It is worth mentioning that after this alteration, the constraints for all the genes are fixed, that is there are no unknown genes left at this stage. For each such constraint, it uses the SiNeC algorithm to construct the network topology. It then only accepts the alteration with the best result. Holistic optimization algorithm describes this process in detail. It consists of following two steps.

**Step 1: Initialization.** It first sets the constraints of all unknown genes to noncritical. Then it uses these constraints to construct the network with minimum distance to the reference and maintains the resulting distance (Lines 2-6).

**Step 2: Climbing.** This step is of significance. It iterates over the set of all the unknown genes. For each such gene *g*_*i*_, it first temporarily sets *g*_*i*_ to critical that is the constraint *c*_*i*_=1. Then it uses this new constraint vector *C* and the given reference network *G*_*R*_ as the guide to construct a new network *G*_*i*_ by applying SiNeC (Line 11). After temporarily altering the constraints for all unknown genes, it chooses the network *G*_*m*_ with the least distance to the reference *G*_*R*_ (Line 14). If the distance between *G*_*m*_ and *G*_*R*_ is better than the current best result, it decides the constraint of the gene *g*_*m*_ should be critical (Line 15-17). Otherwise, it concludes that no single constraint alteration can improve the result and simply returns the current best result (Line 19).

Here, we analyze the performance of the holistic optimization algorithm. The most time consuming step in this algorithm by far is the network construction step (Line 11) using SiNeC. We denote the number of unknown genes with *n*. This step is *O*(*n*^2^).



### Prioritized optimization algorithm

Holistic optimization algorithm carefully tries to construct the network close to the reference network. However, trying *O*(*n*^2^) alternative constraint combinations is prohibitively time consuming as *n* and the network size grow. In this section, we developed a method that alleviates this problem by reducing the number of alterations in the constraint vector.

Our next algorithm utilizes the distance between the network *G*_*i*_ and the reference network *G*_*R*_ which is obtained after altering the constraint of gene *g*_*i*_ to 1 at a time. With these distances, it prioritize the role of gene *g*_*i*_ in the network, i.e., whether gene *g*_*i*_ is critical or not. Smaller values of *dist*(*G*_*i*_,*G*_*R*_) indicate higher likelihood of being critical gene for gene *g*_*i*_ in the target network. Prioritized optimization algorithm describes this idea in detail. Similar to holistic optimization, it also consists of two steps.

**Step 1: Initialization.** Same as holistic optimization, this step starts by initializing the constraints of all the unknown genes to noncritical. It constructs the network and maintains the distance to the reference (Lines 2-6). Then for each unknown gene *g*_*i*_, it temporarily alters its constraint to critical (i.e., *c*_*i*_=1), constructs a new network *G*_*i*_ and keeps the distance *dist*(*G*_*i*_,*G*_*R*_) in *Dist*[*i*] (Lines 7-12).

**Step 2: Climbing.** This step presents the major difference between our two methods. Unlike holistic optimization, the prioritized one iterates over only a subset of unknown genes instead of the whole set. Let us denote this subset with *U*^′^ (Line 16). This subset consists of unknown genes with the smallest value of *Dist*[*i*] obtained in the first step, and it is likely that there are more than one with the same smallest value. For each unknown gene *g*_*i*_ in the set *U*^′^, prioritized optimization temporarily sets it as critical, constructs a network *G*_*i*_ using the new constraint vector *C*, and computes the distance with the reference *dist*(*G*_*i*_,*G*_*R*_) (Lines 17-21). It finalizes the constraint that provides a better result than the current best and continues this process iteratively (Lines 22-25). It returns the current best result until there is no single constraint alteration can improve the result.

Like holistic optimization, constructing the network using SiNeC (Line 19) is the most time consuming step of prioritized optimization. We denote the size of unique *dist*(*G*_*i*_,*G*_*R*_) values among all unknown genes with *k* (*k*≤*n*). Then for each unique *dist*(*G*_*i*_,*G*_*R*_) value, there will be *k* different sets *U*^′^. We represent the size of these *k* sets as *n*_1_, *n*_2_, …, *n*_*k*_ ($n = \sum _{i=1}^{k} n_{i}$). Thus, prioritized optimization executes that step $O(\sum _{i=1}^{k} {n_{i}^{2}})$ times. And we expect that when *k* is large and all *n*_*i*_ have similar values, the time complexity of prioritized optimization is significantly better than that of holistic optimization.



### Sorting critical genes

Both of our holistic and prioritized optimization algorithms employ the SiNeC algorithm to construct the network topology when the constraints of all the genes are determined. Recall from the Section ‘Overview of the SiNeC algorithm’ that an important step of SiNeC is to rank the critical genes. SiNeC applies the Sloan algorithm to do this. The Sloan algorithm ranks genes based on their degrees and distances to the reporter gene (See Section ‘Overview of the SiNeC algorithm’). This strategy however fails to capture the causality between the genes in signal transfer and thus leads SiNeC to incorrect network topologies. Figure 1 explains this on a toy example. In this example, nodes *v*_*s*_ and *v*_*t*_ denote the receptor and reporter genes respectively. Assume that nodes *v*_*a*_ and *v*_*b*_ are critical genes according to the given RNAi constraints. Therefore, we need to rank nodes *v*_*a*_ and *v*_*b*_. Intuitively, *v*_*a*_ should appear before *v*_*b*_ as *v*_*a*_ can pass a signal to *v*_*b*_, and they have the same distance to the reporter. However, since *v*_*b*_ has a larger degree than *v*_*a*_, the Sloan algorithm prefers *v*_*b*_ to come before *v*_*a*_ for a signal starting from the receptor. This causes many redundant edge insertions and deletions (e.g., it requires inserting an edge from *v*_*s*_ to *v*_*b*_). More importantly, it results in an incorrect network topology. In summary, the Sloan algorithm is not tailored for signaling network construction and better ranking algorithms are needed. Next, we develop a new gene ranking algorithm named *Topological Sorting for General Graph (TopSoG)*.

The TopSoG algorithm (see Algorithm 3) is loosely based on the classical topological sorting algorithm [26], which is designed only for directed acyclic graphs (DAGs). A reference network in our problem however may contain cycles. To tackle this problem, we convert the reference network *G*_*R*_=(*V*,*E*_*R*_) to a DAG $\phantom {\dot {i}\!}{G_{R'}} = (V', E')$. Initially, we set $\phantom {\dot {i}\!}{G_{R'}}$ to be the same as the reference network *G*_*R*_. We then update both *V*^′^ and *E*^′^ using the following strategy to convert it to a DAG. We start by applying the Kosaraju’s algorithm [26] to find the *Strongly Connected Components (SCC)* in *G*_*R*_ (Line 2). Let us denote the *i*th SCC with *S*_*i*_. Each *S*_*i*_ defines a small subnetwork in *G*_*R*_ which contains the nodes in *S*_*i*_ and the edges incident to them. We compress each *S*_*i*_ and replace it with a single node in $\phantom {\dot {i}\!}{G_{R'}}$. For each *S*_*i*_, if there is an incoming edge (*u*,*v*) where *u*∈*V*∖*S*_*i*_ and *v*∈*S*_*i*_, we call *v* an *entry point* to *S*_*i*_. Note that there can be multiple incoming edges to *S*_*i*_ leading to possibly multiple entry points. Among all these entry points, we designate one as the *entrance* to *S*_*i*_ whose sum of distances to all the other entry points is the smallest (Lines 5-6). After selecting the *entrance* for every SCC, we replace each SCC with a single node, called *super node* using the strategy below. We first remove all the nodes in *S*_*i*_ from *V*^′^ along with the edges incident to them from *E*^′^. We then insert a new super node *s*_*i*_ into *V*^′^. For each edge (*u*,*v*)∈*E*_*R*_ with *u*∈*V*∖*S*_*i*_ and *v*∈*S*_*i*_, we insert the edge (*u*,*s*_*i*_) into *E*^′^. Similarly, for each edge (*u*,*v*)∈*E*_*R*_ with *v*∈*V*∖*S*_*i*_ and *u*∈*S*_*i*_, we insert the edge (*s*_*i*_,*v*) into *E*^′^. We repeat this process for each *S*_*i*_. The resulting network $\phantom {\dot {i}\!}{G_{R'}}$ is guaranteed to be a DAG (Line 7). We are now ready to rank the nodes.

In the ranking step, we first get the topological ranking *R* of all the nodes in $\phantom {\dot {i}\!}{G_{R'}}$ using the Depth-first-Search (DFS) algorithm in the order they are visited starting from *v*_*s*_ (Lines 10-11). Notice that some of the nodes in this ranking are super nodes. Thus they actually represent a set of nodes which still needs to be ranked. To do that, we run DFS on the subnetwork *S*_*i*_ starting from the entrance node *u*_*i*_, rank the nodes in *S*_*i*_ in the order that they are visited, and replace *s*_*i*_ with the ranked list of nodes in *S*_*i*_ (Lines 14-16). We repeat this for each super node *s*_*i*_ in *R* and obtain a complete ranking of all the nodes in the original reference network *G*_*R*_. Then we extract the ranking of all the critical nodes from *R* (Line 19).

Finally, we emphasize that the DFS strategy used in our algorithm differs from the classical DFS algorithm [26]. When there are multiple unvisited successors, instead of arbitrarily selecting one to traverse next, we select the successor as follows. Consider a possible successor node *v*. We denote the distance between *v* and the source node *v*_*s*_ in the original reference network *G*_*R*_ with *d*_*s*_. Similarly, we denote the distance between *v* and the target node *v*_*t*_ with *d*_*t*_. Among all the unvisited successors, we select the one with the largest (1/*d*_*s*_−1/*d*_*t*_) value, which indicates it is close to *v*_*s*_ but far from *v*_*t*_.



## Results and discussion

In this section, we evaluate the performance of our methods extensively on both synthetic and real datasets. We compute the performance of our methods in terms of the quality of the results and the running time. Next we introduce the datasets and the quality measures used in our experiments and the implementation details.

**Datasets** We use both synthetically generated and real datasets in our experiments. In the following, to simplify our notation, we use the *size* and *density* of the network to represent the number of nodes and the number of edges per node in a network respectively.

*Synthetic dataset.* We run experiments on synthetic networks to observe the performance of our methods under diverse parameters including network size, mutation rate (noise) etc. We randomly generate *scale-free* synthetic networks following the Barabási-Albert model [27] by varying the network size. This model is commonly used in the literature for simulating the real biological network behavior. Using this model, we generate target networks with various sizes 50, 75, 100 and 125. In particular, we generate 10 random networks with density three for each network size. Thus, the dataset contains 40 (i.e., 4 × 10) target networks. According to the problem definition, we impose a receptor and reporter gene pair, RNAi constraints for the gene set on the target network. For each target network, we choose the receptor and reporter genes in the following way. We first find all the shortest paths between all pairs of genes. Among these paths, we choose the longest one as the diameter of the network. Then we set the source node on this path as the receptor gene, and the sink node as the reporter gene. If there are more than one path that can be chosen as the diameter of the network, we choose one of these paths randomly. Upon choosing the receptor and reporter gene pair, we set all the *articulation points* which appear on all the paths from the receptor gene to the reporter gene as the critical genes, and the remaining genes as noncritical.

Each target network has 7 reference networks that are obtained by performing specific level of topological perturbations on it. To do this, we apply the degree preserving edge shuffling method [28] with a given mutation rate (i.e, noise). Specifically, we use seven linearly spaced mutation rates of 5 %, 10 %, …, 35 %. Thus, in total 280 (i.e., 7 × 40) reference networks are created. A mutation rate of *r* means that *r*×|*E*| edges in the target network are shuffled to generate a reference network.

*Real dataset.* This dataset consists of five Wnt signaling networks in the KEGG database. Specifically, they are from organisms *Bos mutus* (*bom*), *Python bivittatus*(*pbi*), *Pan paniscus* (*pps*), *Xenopus laevis*(*xla*), and *Mus musculus* (*mmu*).

**Quality measures.** We use various quantifiable measures to evaluate the performance of our method. We first report the distance between the inferred network *G* and the reference network *G*_*R*_, *dist*(*G*,*G*_*R*_). This criteria measures how well our method constructs the network. Smaller values of this measure indicate better results. We have described this distance criteria formally in Definition 3. We then report the *F-score* in terms of the accuracy of the result compared to the real network topology. This criteria measures how successfully our method build true biological network topology. Larger values of this measure indicate better results. It is worth mentioning that only if the true result is known, we can calculate F-score to measure the result. Next we describe the method to compute F-score.

*F-score.* F-score considers *precision* and *recall* to evaluate the accuracy of the result. We define them with the *true positive (TP)*, *false positive (FP)*, and *false negative (FN)* terms. We calculate the *precision* as $\frac {TP}{TP + FP}$ and *recall* as $\frac {TP}{TP + FN}$. Thus, we calculate the *F-score* as

$$\text{F-score} = \frac{2 \times precision \times recall}{precision + recall} $$

**Implementation details & environment.** We implemented the holistic optimization and prioritized optimization algorithms using Java. We conducted all the experiments on a Linux server which has AMD Opteron dual core processors (up to 2.2 GHz) and 3GB RAM.

**Default parameter settings.** To observe how robust our methods are on the synthetic dataset, we vary a broad spectrum of parameters, such as network size, noise and the number of unknown genes. Notice that the topology of the reference network is affected by the network size and the noise level, and the inference method is affected by the number of unknown genes. In our experiments, unless stated otherwise, we always set the default values for these three parameters as follows: network size (100), noise level (15 %), the number of unknown genes (15).

### Effects of parameters on the inference methods

To test the robustness of our methods under various parameters, we run experiments on synthetic dataset and compute the accuracy of results. In this respect, we vary the following three parameters: (i) network size, (ii) noise, and (iii) the number of unknown genes in the network. To observe the impact of each parameter on our methods, each time we only vary one parameter and fix the other parameters to their default values. To ensure the results are reliable, for each parameter, we conduct experiments on 10 reference networks and report their average distance *dist*(*G*,*G*_*R*_) and running time.

**Effect of network size.** First, we explore the impact of network size. We fix the noise to 20 % and the number of unknown genes to 15. We experiment for network size 50,75,100 and 125.

**Fig. 2:**
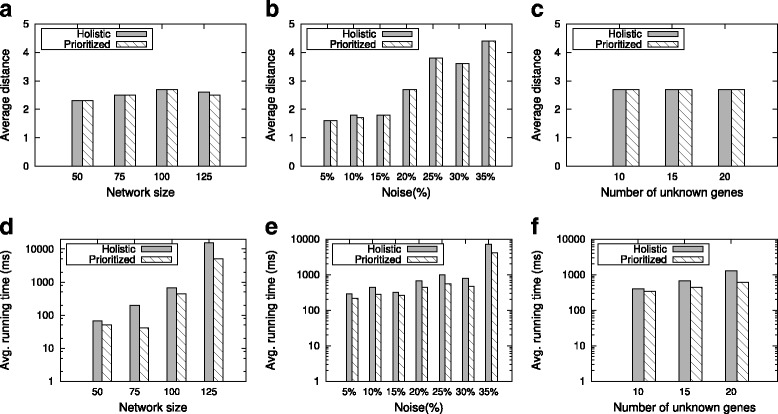
Effect of parameters on the inference methods. **a**, **b**, and **c** show the average distance between the constructed and the reference networks for varying network size, noise and number of unknown genes respectively. **d**, **e**, and **f** show the running time of the inference methods for the same setup. The running time is reported in milliseconds (ms) and presented in log-scale

For all different network sizes, we observe that our two methods both successfully build a network topology which is close to the reference network (Fig. 2a). Generally, both of them obtain roughly same distance values. Thus, in regards to the quality of the results, no clear winner emerges. On the other hand, we also observe that the distance between *G* and *G*_*R*_ lightly grows as the network size increases. This is because when the noise and density are set, the increase of the network size leads to the increase of the number of edges shuffled.

The running time of our methods is significantly fast (Fig. 2d). Even for networks with 125 nodes, the running time is only around 10 seconds. For all network sizes, we observe that the prioritized optimization runs faster than the holistic optimization method. This is expected since the former one tests fewer constraint combinations. Moreover, we also see that the running time of each inference method grows with the network size. This is because the number of edges in the reference network contributes a lot to the complexity of our methods. As the number of nodes grows, the number of edges in the reference networks also grows when the network density is fixed.

**Effect of noise.** Next, we consider the impact of noise. We set the network size to 100 and the number of unknown genes to 15. We experiment for noise 5 *%*,10 *%*,…,35 *%*.

For all noise values, in terms of the distance between *G* and *G*_*R*_, we observe similar results with those in (Fig. 2a and b). Generally, the resulting distance values are roughly same. Both methods successfully build a network topology close to the reference network. On the other hand, we also observe that the distance increases with the increase in noise. This is because when the noise grows, the amount of deviation between the reference and the target network will also increase. Thus, in order to be consistent with the

RNAi constraints, more edge insertions and deletions are expected to happen in the reference network.

The running time of our methods is very fast (in milliseconds to seconds) (Fig. 2e). For all noise values, we see that compared to the holistic optimization, the prioritized one runs faster. Moreover, we also observe that the running time increases as the noise level increases. One possible reason is that with the growth of the difference between the reference and the target network, more time is needed to reach the smallest distance value.

**Effect of the number of unknown genes.** Finally, we focus on the impact of the number of unknown genes. We fix the network size to 100 and the noise to 20 %. We experiment for the number of unknown genes 10, 15 and 20.

For all numbers of the unknown genes, like our previous experiments, we observe the similar distance results (Fig. 2c). Both methods have a small distance value. Interestingly, as the number of unknown genes increases, we see that the distance values do not noticeably change. Thus, our methods are robust to the change of the number of unknown genes.

Similar to our other experiments, our methods demonstrate practical running time (Fig. 2f). Both methods construct networks from milliseconds to seconds. We observe that the advantage in running time of the prioritized optimization does not change. Moreover, we also see that the running time increases gradually with the number of unknown genes, which is very favorable since there are usually many unknown genes in practical applications.

In summary Our experiments show that our methods are robust to various parameters. Under a variety of parameter settings, both the holistic and prioritized optimization successfully infer a network topology with a small distance to the reference network. Among all three parameters, we observe that the fitness between the predicted and actual network is affected most by the noise level. Although both methods yield similar distance values, the prioritized optimization runs much faster. Moreover, we also observe that the network size affects the running time of two methods the most. The running time grows with the network size. According to the above discussion, we conclude that the prioritized optimization is more desirable since it obtains the similar distance value as the holistic one in a much faster time. As a result, we apply the prioritized optimization in the remaining experiments.

### Ranking strategies: Sloan vs. TopSoG

Existing methods [20] such as SiNeC use the Sloan algorithm [25] to rank the critical genes in the network. We have already discussed how the Sloan algorithm works (Section ‘Overview of the SiNeC algorithm’), its limitations, and developed a new ranking algorithm named TopSoG (Section ‘Sorting critical genes’). Here, we seek the answer to the question whether TopSoG indeed yields any improvement experimentally. We fix the network size and noise to 100 and 20 % respectively and vary the number of unknown genes from 10 to 20. We compare the performance of our prioritized optimization in terms of the distance between the constructed and the reference networks and the running time when it employs Sloan and TopSoG algorithms.

**Fig. 3:**
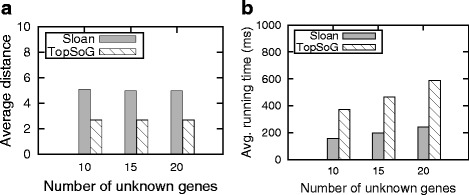
Comparison of the Sloan and TopSoG ranking strategies. **a** shows the distance between the inferred and the reference networks. **b** reports the running time of the inference algorithm when employed with each strategy in milliseconds (ms)

Figure 3a presents the average distance between the constructed and the reference networks. We observe that TopSoG is superior to Sloan in minimizing the distance values regardless of the number of unknown genes. However, this improvement comes with a price of an increase in the running time. Figure 3b shows the running time of our prioritized optimization for both ranking strategies. We see that on the average the Sloan is faster than TopSoG in all cases. That said, both strategies have practical running times as they both work in less than a second. *Thus, we conclude that the TopSoG algorithm is more preferable as the accuracy of the network topology is of primary target in network construction.* In the rest of our experiments, we use TopSoG to rank critical genes.

### Comparison with the exhaustive search method

As mentioned before, our inference methods employ a heuristic strategy which greedily determine the role of next unknown gene. It is interesting to see how well our methods perform comparing to the deterministic exhaustive approach, which takes all possible combinations of unknown genes into account. To answer this question, we conduct a set of experiments with the synthetic dataset. We change the number of unknown genes from 10 to 20 with the network size and noise fixed as 100 and 20 % respectively. For each number of unknown genes, we repeat the experiment with 10 reference networks and compute the average.

**Fig. 4:**
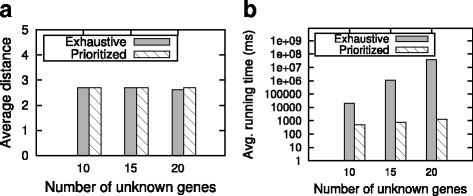
Comparison of the prioritized and the exhaustive methods. **a** shows the average distance between the inferred and reference networks. **b** reports the running time in milliseconds (ms)

For all numbers of unknown genes, our method obtains a high accuracy (Fig. 4a). Although our method is heuristic, it obtains similar or even exactly the same distance values as the optimum results produced by the exhaustive approach.

Besides the accuracy, we also pay attention to the efficiency of our method. We observe that in terms of running time, our method has great advantage (Fig. 4b). As the number of unknown genes grows, the running time of our strategy grows only quadratically while that of the exhaustive search is exponential (we discuss about the time complexity in Section ‘Prioritized optimization algorithm’). Thus, when the networks are large or with great number of unknown genes, using exhaustive strategy is impractical, whereas it only takes negligible time for our method to produce almost the same quality results as the exhaustive strategy.

### Evaluations on real dataset

In the above sections, we have demonstrated the robustness of our method under various parameters. Even though the Barabási-Albert model is used to simulate the behavior of the real biological networks, slight differences might exist between the resulting and real network topological characteristics. To show the applicability of our method to real networks, in this section, we evaluate our method with a real dataset. Networks in this dataset are from the following organisms, *Bos mutus* (*bom*), *Python bivittatus*(*pbi*), *Pan paniscus* (*pps*), *Xenopus laevis*(*xla*), and *Mus musculus* (*mmu*). We set *xla* and *mmu* to the target networks, and the rest are taken as references. When two organisms are orthologs, we say that a node (gene) in one network has a corresponding node in another, but it is possible to have nodes not matching between two organisms. If a node is absent in the target network, we remove it and its incident edges in the reference network. We change the amount of unknown genes *n* from 4 to 20. For each *n* value, we set the constraints of *n* randomly picked genes from the target gene set to “unknown”. According to the network’s topology, we decide the roles of the remaining nodes, i.e., whether it is critical or not. To ensure the results are reliable, for each parameter, we conduct the experiment for 200 times and compute the average *F-score* of the resulting network.

**Fig. 5:**
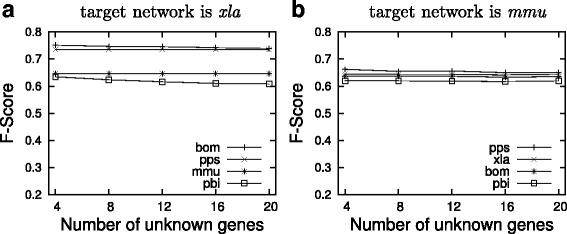
The F-score of the constructed Wnt signaling network using different reference networks. **a** shows the F-score for target network *xla*. **b** shows the F-score for target network *mmu*

First, we fix *xla* as the target network and the rest as the reference. We set *nemo-like kinase* (KEGG entry: xla398295) and *glycogen synthase kinase 3 beta* (KEGG entry: xla399097) as the receptor gene and the reporter gene respectively. As Fig. 5a shows, the F-score of resulting topology is as high as 0.75 when *bom* or *pps* is the reference network. If *mmu* or *pbi* is the reference network, the accuracy drops slightly but still remains significantly high, which indicates that the choice of the reference impacts the accuracy of the result. Moreover, we find that the accuracy of our method is robust as the number of unknown genes grows. This is very promising since we expect to have many unknown genes in real networks, especially for those less studied organisms.

Then we fix *mmu* as the target network. We set *nemo-like kinase* (KEGG entry: mmu18099) and *naked cuticle 2 homolog* (KEGG entry: mmu72293) as the receptor gene and the reporter gene respectively. We make the similar observation that our method is robust to the growing number of unknown genes while having a high accuracy (Fig. 5b).

When the rest of the organisms are target networks, we observe the similar results (results not shown). Last, we turn our attention to the running time of our method. In this dataset, each network is inferred within less than 100 ms. In summary, our method is a practical tool for constructing real signaling networks because of its efficiency and high accuracy.

## Conclusions

In this study, we presented two novel methods for constructing signaling networks with incomplete RNAi data under the guidance of a reference network. These methods infer the network topology, which is consistent with the RNAi experiments and is close to a given reference network. We also presented a new biologically relevant gene ranking method for signaling network construction. Our experiments showed that the new ranking strategy greatly improve our methods in minimizing the distance to the reference. Moreover, both of our methods construct highly accurate signaling networks in a much faster time than an exhaustive research. We observed that although the accuracy of our two methods are comparable, the prioritized optimization method outperforms the holistic method in terms of the running time. Application of our method to the real Wnt signaling network demonstrated its efficiency and applicability in real signaling networks.
